# External evaluation of a deep learning-based algorithm for automated lung tumor and esophagus segmentation on CBCT

**DOI:** 10.1088/2057-1976/aeaf5c

**Published:** 2026-10-14

**Authors:** Zhehao Zhang, Dean Hobbis, Jue Jiang, Aneesh Rangnekar, Chloe Choi, Michael Waters, Harini Veeraraghavan, Geoffrey Hugo

**Affiliations:** 1 Department of Radiation Oncology, WashU Medicine, St. Louis, MO, United States of America; 2 Department of Medical Physics, Memorial Sloan Kettering Cancer Center, New York, NY, United States of America

**Keywords:** adaptive radiotherapy, lung cancer, deep learning, auto-segmentation, CBCT

## Abstract

Online adaptive radiotherapy requires fast and accurate delineation of targets and organs at risk on cone-beam computed tomography (CBCT). Although deep learning (DL)-based CBCT auto-segmentation methods have been proposed, their clinical generalizability remains insufficiently validated. This study aimed to externally evaluate a pretrained DL model for lung tumor and esophagus segmentation on CBCT. Originally trained using C-arm linac CBCT images, the model was directly applied to an independent cohort of 12 lung cancer patients, each with 5 CBCT images acquired at a separate institution on an O-ring linac. Rigid propagation of planning CT contours served as a minimal baseline to determine whether the model matched or outperformed conventional rigid registration for gross tumor volume (GTV) and esophagus segmentation using geometric and dosimetric measures. Compared with rigid contour propagation, the model significantly improved GTV segmentation accuracy, with mean Dice similarity coefficient (DSC) increasing from 0.66 to 0.76. Significant differences in GTV D98% were observed between the model-generated contours and the reference contours (50.69 vs 53.58 Gy). The model showed a statistically non-significant decrease in performance compared with previously reported results on its original testing dataset (mean DSC = 0.84), which shared the same distribution as the training dataset. For esophagus segmentation, evaluation of 26 scans from 6 patients showed that the model achieved geometric accuracy within peri-target contour rings comparable to rigid propagation, with mean DSC of 0.72 and 0.70, respectively. Similar esophagus V32Gy values were observed between the model-generated and reference contours (0.06 vs 0.06 cc). Significantly reduced accuracy was observed compared with previously reported results, with mean DSC decreasing from 0.79 to 0.72. These findings highlight that DL-based CBCT auto-segmentation may suffer performance degradation under domain shifts across patient populations, clinical workflows, and imaging systems, emphasizing the necessity of external evaluation beyond the original development environment.

## 1. Introduction

Stereotactic body radiation therapy has proven to be an effective treatment option for non-small cell lung cancer (NSCLC) (Buchberger and Videtic 2023). Despite its clinical success, challenges remain regarding dose escalation and the risk of delivering ablative doses to adjacent organs at risk (OARs), especially in the treatment of central and ultra-central lung tumors (Timmerman *et al* 2006, Bezjak *et al* 2019). Online adaptive radiotherapy (ART) addresses this issue by adapting the treatment plan prior to each fraction based on patient’s anatomy while the patient remains on the treatment table, enabling smaller planning target volume (PTV) margins while maintaining target coverage and minimizing OAR toxicity (Qin *et al* 2013). Online ART requires fast and accurate delineation of targets and OARs on daily cone-beam computed tomography (CBCT). However, the inferior image quality and limited tissue contrast of CBCT make manual contouring both time-consuming and susceptible to errors and interobserver variability, underscoring the importance of reliable auto-segmentation in online ART.

Although deep learning (DL) methods have been successfully applied for direct auto-segmentation on CT images (Yang *et al* 2018, Bi *et al* 2019), extending these approaches to CBCT presents substantial challenges. Previous studies have explored direct DL segmentation (Léger *et al* 2020, Schreier *et al* 2020, Dahiya *et al* 2021), deformable image registration (DIR)-based contour propagation (Zambrano *et al* 2013, Zhen *et al* 2013, Han *et al* 2021, Liang *et al* 2023a), and hybrid approaches that combine both strategies (Beekman *et al* 2022, Jiang and Veeraraghavan 2022, Liang *et al* 2023b). While direct segmentation is limited by the inherently poor image quality of CBCT and the scarcity of annotated training data, DIR-based methods tend to bias toward the original planning CT (pCT) contours and may fail in the presence of large anatomical variations between scans (Kurz *et al* 2016). Hybrid methods seek to leverage the complementary strengths of both approaches. Specifically for the thoracic CBCT segmentation, Jiang and Veeraraghavan (2022) developed a patient-specific anatomic context and shape prior aware registration-segmentation model (PACS), which jointly optimized a registration network and a segmentation network, enabling the two tasks to guide each other. PACS demonstrated the ability to generate reasonably accurate segmentations for lung tumors as well as the highly deformable esophagus on conventional C-arm linac CBCT, making it a practical and representative hybrid framework for CBCT auto-segmentation.

Recently, the Ethos O-ring linac platform equipped with the HyperSight CBCT imaging system (Varian Medical Systems) has enabled rapid CBCT acquisition with enhanced image quality and streamlined online ART through an on-board artificial intelligence (AI)-driven treatment planning system (TPS) (Schiff *et al* 2022a, 2022b). These features make Ethos a suitable platform for CBCT-based online ART, thereby suggesting that auto-segmentation methods developed and validated on this system to assist ART would hold greater clinical relevance.

However, the training and evaluation datasets used in most existing CBCT auto-segmentation studies, including PACS, were limited to CBCT images acquired using conventional gantry-mounted imaging system on a C-arm linac, such as on-board imager (OBI) on the Varian TrueBeam system. Given the well-known issue that DL models can experience substantial performance degradation when tested on out-of-distribution (OOD) data, where the testing data deviates from the training data distribution (Jin *et al* 2024), it is both necessary and clinically meaningful to conduct external evaluations to validate model generalizability across different patient populations and health systems with diverse imaging platforms, clinical workflows, and physician practice patterns prior to clinical adoption.

Therefore, this study aims to provide an external evaluation of PACS by directly applying the pretrained model, initialized from the publicly available checkpoint trained on conventional C-arm linac (TrueBeam) CBCT, to O-ring linac (Ethos) CBCT acquired from an independent institution using a different imaging system and contoured by different physicians. Its generalizability for GTV and esophagus segmentation across different clinical environments was assessed by analyzing the geometric and dosimetric deviations between the predicted and manually edited reference contours.

## 2. Methods

### 2.1. Study cohorts

The original PACS model was trained on a dataset consisting of 56 patients with locally advanced NSCLC, comprising cases from the authors’ internal institutional cohort and an open-source dataset. For each patient, one 4D-pCT and up to six 4D-CBCTs were available, while only mid phase CT and CBCT images were utilized for training. All CBCT images were acquired using the standard OBI CBCT scanner on a TrueBeam system. Additional details of the training dataset can be found in the original PACS paper (Jiang and Veeraraghavan 2022).

With the Institutional Review Board approval, this retrospective evaluation study included 12 patients with central or ultra-central early-stage (stage I–IIA) NSCLC (Howington *et al* 2013) from an independent cohort that was distinct from the training dataset. All patients received five-fraction radiation therapy delivered using the Ethos linac and its online adaptive TPS. The detailed demographic differences between patients included in training of the published model and the current evaluation cohorts are shown in table 1.

**Table 1.** Data distribution for training and external evaluation datasets.

Characteristic		Original training dataset (*n* = 56)	External evaluation dataset (*n* = 12)
Sex			

	Male	38	2
	Female	18	10

Tumor size			

	Small (⩽5cc)	5	5
	Medium (5–10cc)	3	1
	Large (>10cc)	48	6

Tumor location			

	Superior	22	7
	Middle	24	1
	Inferior	10	4

### 2.2. Adaptive workflow and evaluation data

For the evaluation patient cohort, pCT images were acquired for initial treatment planning, with an in-plane resolution ranging from 0.98 × 0.98 to 1.27 × 1.27 mm^2^ and a slice thickness of 2–3 mm. Tumor and OAR contours were delineated by the treating radiation oncologist and/or dosimetrist, with PET-CT image fusion incorporated at the discretion of the radiation oncologist’s discretion. The gross tumor volume (GTV) served as the basis for defining the treatment target, from which the PTV was generated by applying a 5 mm isotropic margin. Initial treatment plans were created for all patients with a prescription dose of 55 Gy to the PTV, with an acceptable variation of planning 45 Gy to the GTV.

Prior to each treatment, an end-exhale breath-hold CBCT was acquired on the Ethos platform with HyperSight scanner. The initially reconstructed CBCTs generally had smaller in-plane pixel sizes (0.96 × 0.96–1.05 × 1.05 mm^2^) than the pCT, with 44–120 slices at a thickness of 2 mm. The Ethos system automatically generated OAR contours for structures near the target (Archambault *et al* 2020), and our institutional workflow rigidly propagated the target contours from the pCT to the CBCT. As part of the standard Ethos online adaptive workflow, a predefined contour ring was automatically generated for each CBCT by expanding the PTV by 3 cm in the axial direction and 1.2 cm in the superior–inferior direction. Only the target contours and the portions of OAR contours within this contour ring were then manually corrected and confirmed by the radiation oncologist to reflect the patient’s anatomy-of-the-day. The resulting edited contours served as the reference for daily CBCTs. All segmentation evaluations in this study were restricted to the contour ring, as only contours within this region had been clinically reviewed and approved. In addition, cases with esophageal volumes under 0.5cc within the contour ring were excluded to minimize instability in the evaluation results. Consequently, a total of 60 CBCT cases were included for GTV segmentation evaluation, while 26 cases from 6 patients were included for esophagus segmentation evaluation.

Each patient was provided with two plans: a reference plan, which directly projected the initial plan onto the patient’s daily anatomy, and an adapted plan, which was generated using edited reference contours and re-optimization. The expected 3D dose distributions were calculated and compared between the two plans, and the superior plan was selected for treatment. Specifically, the adapted plan was deemed superior if it resolved at least one OAR hard constraint and/or achieved an improvement in PTV or GTV coverage over 5%.

### 2.3. Auto-segmentation model

The pCT and daily CBCT images were resampled to the same voxel spacing and rigidly registered using the method provided by PACS^3^3Source code available at: https://github.com/The-Veeraraghavan-Lab/OneShotPACS.. The PACS model then utilized the patient-specific pCT and corresponding delineations as anatomic context and shape priors to assist the CBCT segmentation. Details of the model design and development were described in the prior work by Jiang and Veeraraghavan (2022). Briefly, PACS made use of a jointly trained recurrent registration network and segmentation subnetworks. The model sequentially refined the DIR between rigidly aligned pCT and CBCT images pairs and integrated the resulting registration information to generate the final segmentation. Since the PACS model focused on auto-segmentation of the GTV and esophagus, only their contours were evaluated in this study, despite the availability of additional OAR contours in our dataset.

### 2.4. Geometric evaluation metrics

To be consistent with the PACS study, geometric variabilities between the automatically delineated contours and the corresponding reference were quantified using the Dice similarity coefficient (DSC), surface DSC (sDSC), and 95% Hausdorff distance (HD95). Assuming the volumes defined by the auto-delineation and the reference contours for a particular structure are denoted as *A* and *B*, respectively, the DSC measures their overlap over the sum of their total volume:

\begin{align*}{\mathrm{DSC}}\left( {A,{ }B} \right) = \frac{{2\left| {A{{\mathop \cap \nolimits}}B} \right|}}{{\left| A \right| + \left| B \right|}}.\end{align*}

While DSC can assess the volumetric overlap of two contours, sDSC evaluates the overlap of two surfaces at a specified tolerance *τ* to focus on clinically relevant boundary errors (Nikolov *et al* 2018):

\begin{align*} {{\mathrm{sDSC}}\left( {\partial A,\partial B} \right) = \frac{{\left| {\partial A{{\mathop \cap \nolimits}}\mathcal{B}_B^{\left( \tau \right)}} \right| + \left| {\partial B{{\mathop \cap \nolimits}}\mathcal{B}_A^{\left( \tau \right)}} \right|}}{{\left| {\partial A} \right| + \left| {\partial B} \right|}},} \end{align*}

where ∂A and ∂B denote the surfaces of *A* and *B*, respectively. The sets $\mathcal{B}_A^{\left( \tau \right)} = \{ x \in {\mathbb{R}^3}|\exists \tilde x \in \partial A,x - \tilde x \unicode{x2A7D} \tau \} $ and $\mathcal{B}_B^{\left( \tau \right)} = \left\{ {x \in {\mathbb{R}^3}{\mathrm{|}}\exists \tilde x \in \partial B,x - \tilde x \unicode{x2A7D} \tau } \right\}$ represent the border regions associated with ∂A and ∂B at a given tolerance *τ*, respectively. A tolerance of 4.38 mm, determined based on inter-observer variation in manual segmentations (Jiang and Veeraraghavan 2022), was used for the sDSC calculation.

Compared to the standard HD metric, HD95 measures the surface distance of two contours at 95th percentile rather than the maximum to reduce the sensitivity to outliers:

\begin{align*} {\mathrm{HD95}}\left( {\partial A,\partial B} \right) &amp;= \max \left( {{h_{95}}\left( {\partial A,\partial B} \right),{h_{95}}\left( {\partial B,\partial A} \right)} \right) \nonumber\\ {h_{95}}\left( {\partial A,\partial B} \right) &amp;= \mathop {{\mathrm{percentil}}{{\mathrm{e}}_{95}}}\limits_{a \in \partial A} \left\{ {\mathop {\min }\limits_{b \in \partial B} \left\{ {d\left( {a,b} \right)} \right\}} \right\}, \end{align*}

where $d\left( {a,b} \right)$ represents the 3D Euclidean distance between point a from ∂A and point b from ∂B.

### 2.5. Dosimetric evaluation metrics

The dosimetric evaluation was processed by superimposing the dose distribution of the selected treatment plan onto the reference contours and auto-segmentation contours. Although re-optimization using generated contours may be a preferred approach (Sherer *et al* 2021), only the GTV and esophagus contours were available from the segmentation model. Re-optimizing the treatment plans using only these two updated structures was not expected to produce meaningful changes.

Based on the institutional dosimetric objectives and constraints used during online adaptation (GTV D98% > 45 Gy and esophagus V32Gy < 0.5 cc), the dosimetric parameters evaluated included GTV mean dose (Dmean) and D98%, as well as esophagus Dmean and V32Gy. In addition, the volumes of both the GTV and esophagus within the contour ring delineated by different approaches were calculated.

### 2.6. Statistical analysis

Rigid registration was used as a minimal baseline in this study to evaluate whether PACS matched or exceeded this benchmark. To account for patient-level clustering from multiple CBCT scans per patient, a linear mixed-effects model was used to compare the segmentation accuracy of rigid pCT contour propagation (Rigid-Reg) and PACS auto-segmentation against the reference contours. The segmentation method was modeled as a fixed effect, with patient included as a separate random effect. An additional linear mixed-effects model was used to compare the geometric performance of PACS on the Ethos dataset in the current study with the previously reported reference performance on TrueBeam CBCTs. For all analyses, *p* < 0.05 was considered statistically significant.

## 3. Results

### 3.1. Geometric results

The geometric segmentation accuracies for the GTV and esophagus obtained using Rigid-Reg and PACS are presented in table 2. The previously reported performance of the PACS model on conventional TrueBeam CBCT datasets from nine patients in the original study (Jiang and Veeraraghavan 2022) is included as a reference. The detailed performance of PACS relative to Rigid-Reg, which serves as the starting point for the DL-based segmentation, for each evaluation case is shown in supplementary figure S1.

**Table 2.** Geometric accuracies (mean ± standard deviation) of GTV and esophagus segmentation. Statistical comparisons include Rigid-Reg versus PACS within the current study (Rigid-Reg vs PACS) and PACS performance between the current study and previously reported reference values (Current study vs Reported reference).

		Rigid-Reg (Ethos)	PACS (Ethos)	*p*-value^a^ (Rigid-Reg vs PACS)	Reported PACS reference (TrueBeam)	*p*-value^a^ (Current study vs Reported reference)
GTV	DSC	0.66 ± 0.25	0.76 ± 0.24	<0.001	0.84 ± 0.08	0.337
	sDSC	0.87 ± 0.25	0.92 ± 0.23	0.018	0.98 ± 0.04	0.439
	HD95 (mm)	5.25 ± 4.46	4.49 ± 4.96	0.001	3.33 ± 2.02	0.503

Esophagus	DSC	0.70 ± 0.13	0.72 ± 0.11	0.307	0.79 ± 0.13	0.044
	sDSC	0.97 ± 0.03	0.98 ± 0.03	0.245	0.91 ± 0.12	0.033
	HD95 (mm)	4.20 ± 1.91	3.75 ± 1.13	0.231	3.10 ± 2.16	0.505

Abbreviations: GTV = gross tumor volume; DSC = Dice similarity coefficient; sDSC = surface Dice similarity coefficient; HD95 = 95% Hausdorff distance.

^a^ Linear mixed-effects model (patient as a random effect).

For GTV segmentation, PACS demonstrated significant improvements over Rigid-Reg across all metrics. Although the PACS model achieved a mean DSC exceeding 0.75 for GTV on the Ethos dataset, large deviations were observed across cases and evaluation metrics. Overall, the geometric accuracy on the Ethos dataset was lower than observed in the originally reported dataset across all metrics, although the differences did not reach statistical significance. These results highlight the challenge of providing robust performance when encountering out-of-distribution datasets with imaging characteristics different from those encountered during training.

Regarding esophagus segmentation, no statistically significant differences were observed between Rigid-Reg and PACS, indicating that the PACS model exhibited limited improvement over simple rigid registration on the Ethos dataset and achieved comparable geometric accuracy. Compared with the originally reported results on the TrueBeam dataset, PACS showed significantly degraded esophagus segmentation performance on the Ethos dataset, as reflected by lower mean DSC and higher HD95 values. In contrast, sDSC showed the opposite trend without statistical significance.

### 3.2. Dosimetric results

Table 3 summarizes the comparison of volume and dosimetric metrics derived from contours generated by Rigid-Reg and PACS. For the GTV, both Rigid-Reg and PACS exhibited non-significant under-segmentation. Significant dosimetric deviations from the reference contours were observed in both Dmean and D98%, with PACS demonstrating closer agreement.

**Table 3.** Volume and dosimetric parameters (mean ± standard deviation) for gross tumor volume (GTV) and esophagus segmentation, including statistical comparisons between different auto-segmentation methods with the reference contours.

		References	Rigid-Reg	*p*-value^a^(Rigid-Reg vs Reference)	PACS	*p*-value^a^(PACS vs Reference)
GTV	Volume (cc)	11.62 ± 10.14	11.42 ± 9.90	0.087	11.35 ± 9.70	0.297
	Dmean (Gy)	61.16 ± 1.86	57.86 ± 8.68	<0.001	59.82 ± 4.96	0.006
	D98% (Gy)	53.58 ± 4.56	47.48 ± 12.78	<0.001	50.69 ± 9.35	0.002

Esophagus	Volume (cc)	5.38 ± 3.77	4.38 ± 3.29	0.004	4.16 ± 3.96	0.008
	Dmean (Gy)	16.86 ± 3.20	16.92 ± 3.83	0.831	16.08 ± 2.99	0.382
	V32Gy (cc)	0.06 ± 0.11	0.13 ± 0.29	0.070	0.06 ± 0.15	0.820

a Linear mixed-effects model (patient as a random effect).

In regard to the esophagus, both Rigid-Reg and PACS showed significant under-segmentation inside the contour ring. No significant differences were observed in Dmean and V32Gy between auto-contours and the reference contours, The relationship between each auto-segmented contour and the reference contour with respect to volume and dosimetric parameters for each evaluation case is shown in supplementary figure S2.

### 3.3. Representative auto-segmentation results

Figure 1 shows qualitative examples of CBCT GTV segmentation along with the corresponding DSC values. The red contours represent the reference GTV, while the yellow contours correspond to the auto-segmentation. The purple regions indicate the patient-specific contour rings. In case 1, the PACS model achieved good performance (DSC = 0.87) even though the initial rigidly propagated pCT contour (DSC = 0.53) exhibited an obvious mismatch with the tumor on the CBCT, despite the presence of metal artifacts.

**Figure 1.**
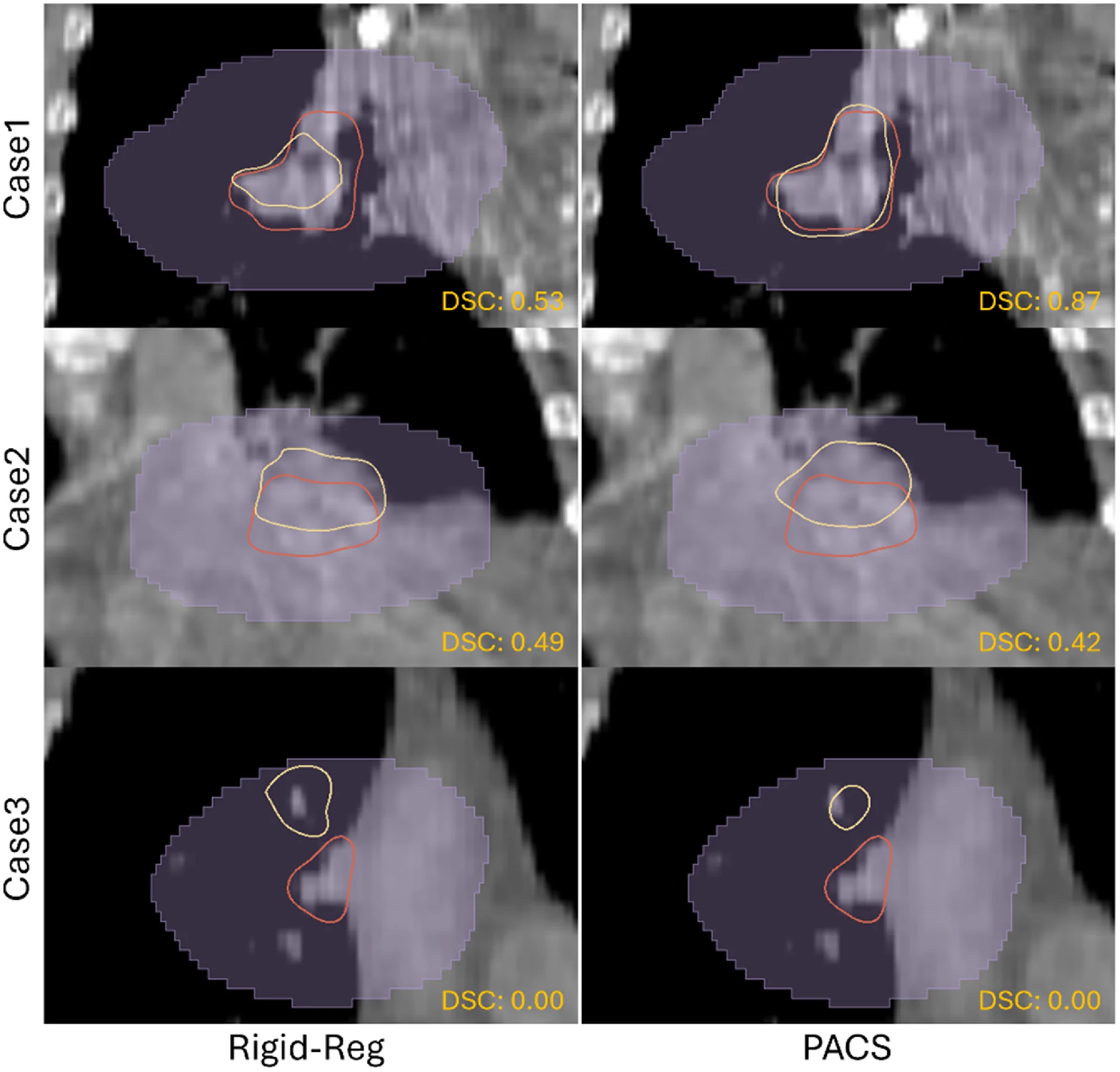
Examples of gross tumor volume (GTV) segmentation generated by Rigid-Reg and PACS. Only the coronal slice is shown for illustration. The purple circles represent the contour rings. Red contours denote the reference GTV boundaries, while yellow contours represent the predicted segmentations.

However, the PACS method failed in case 2, where the image contrast between the tumor and surrounding tissues was too low for PACS to predict accurate segmentations. As a result, PACS only achieved comparable results (DSC = 0.42) compared to the initial Rigid-Reg (DSC = 0.49). In case 3, the Rigid-Reg incorrectly delineated a vessel as GTV (DSC = 0.00), possibly due to anatomical changes between the pCT and CBCT acquisitions. Since the contour propagated from the pCT served as a poor and misleading starting point, the PACS failed to correct it (DSC = 0.00).

Three representative examples of esophagus segmentation are illustrated in figure 2. The reference and auto-segmented esophagus contours are shown in blue and green, respectively. In the first case, the starting point Rigid-Reg achieved a reasonable match with the reference (DSC = 0.70). PACS further improved segmentation accuracy (DSC = 0.82).

**Figure 2.**
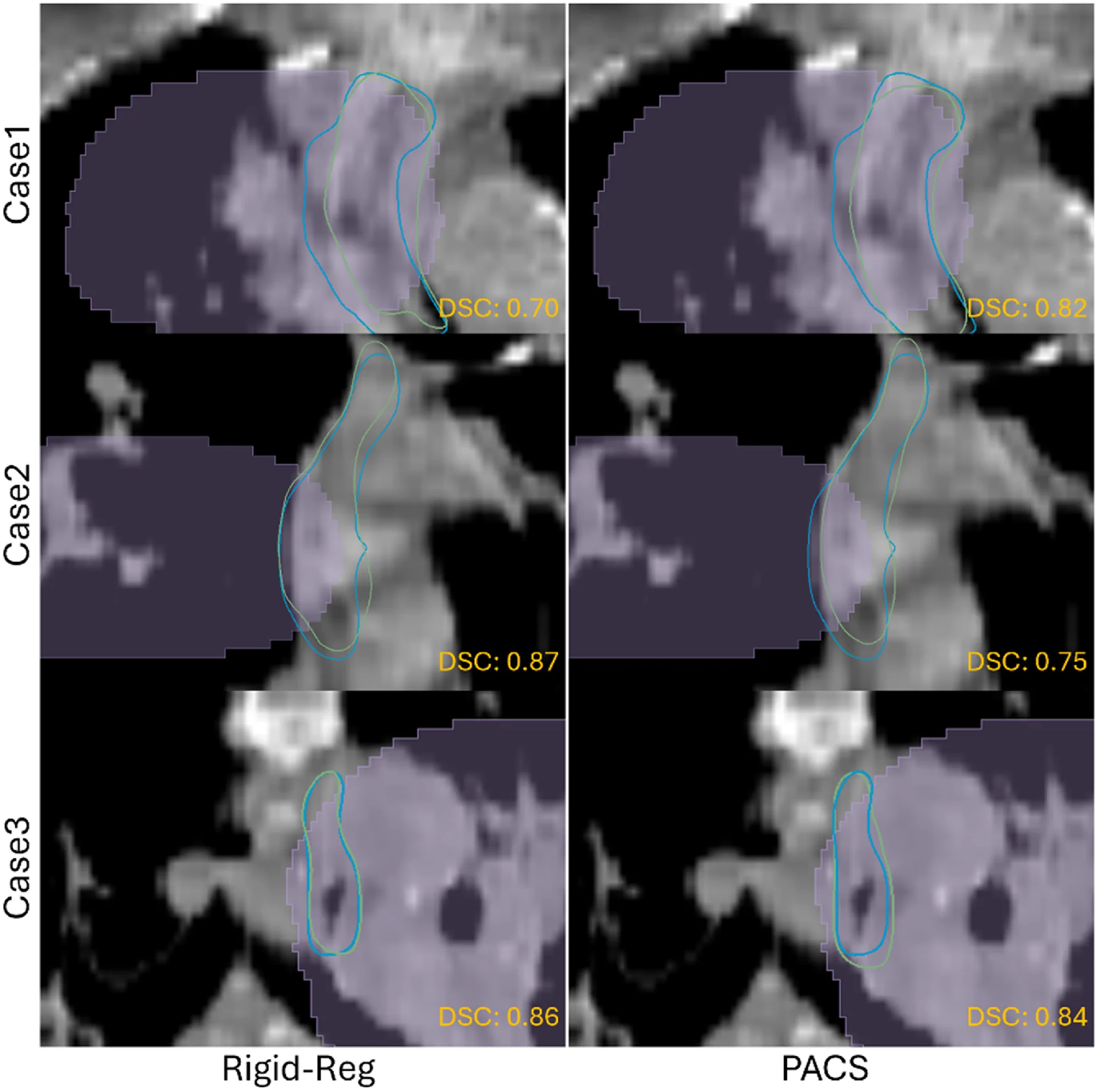
Examples of esophagus segmentation generated by Rigid-Reg and PACS. Only the coronal slice is shown for illustration. Blue contours represent the reference esophagus boundaries, while green contours indicate the predicted segmentations. Only the regions within purple circles (contour rings) were included in the metric calculations.

In the second case, Rigid-Reg achieved a higher overlap with reference (DSC = 0.87), whereas PACS under-segmented the esophagus, leading to a lower DSC (0.75). In the third case, PACS slightly over-segmented the esophagus, whereas Rigid-Reg maintained closer agreement with the reference (DSC = 0.86), leading to better segmentation performance.

## 4. Discussion and conclusion

Due to the poor image quality and the scarcity of delineated CBCT images, the application of DL-based auto-segmentation on CBCT is still challenging, particularly for soft-tissue structures such as tumors and organs like the esophagus that exhibit low contrast and substantial anatomical deformation during treatment. By incorporating DIR-based contour propagation into the auto-segmentation process, the PACS model leveraged the pCT and its corresponding contours as patient-specific anatomic context and shape priors for CBCT segmentation, demonstrating promising accuracy on CBCTs acquired from conventional C-arm LINAC units (TrueBeam). Nevertheless, external validation remains necessary for such auto-segmentation models to assess their generalizability on independent datasets with different patient populations, imaging systems, and clinical practices. This step is essential for translating these models into real-world clinical practice (Collins *et al* 2024).

Varian Ethos is now a commercially available CT-guided radiotherapy platform supporting online ART. Given the differences in imaging geometry, detector panels, and reconstruction algorithms between the CBCT systems integrated on Ethos and conventional LINACs (Cai *et al* 2019), this external evaluation investigated the generalizability of an AI model pretrained on TrueBeam data on the Ethos platform at a different institution. The evaluation was performed under substantial domain shifts from the training environment, including differences in imaging systems, treatment workflows, and institutional practices, with a particular focus on the tumor (GTV) and esophagus segmentation. Although the impact of domain shift on DL-based applications has been widely recognized, external validation of AI models under their intended deployment conditions remains limited. This study provides an example of such an evaluation by assessing a pretrained CBCT auto-segmentation model under real-world deployment conditions, highlighting the importance of rigorous external evaluation in assessing model generalizability across different clinical environments and informing future AI model development with considerations for cross-environment transferability.

For GTV segmentation, the PACS model generally achieved improved geometric accuracy compared with contours rigidly propagated from the pCT. However, this improvement was not consistently observed, particularly in cases with limited image quality. Furthermore, the model may produce grossly inaccurate segmentations under certain conditions, such as incorrect initial contour propagation in regions distant from the reference (figure 1, case 3), representing catastrophic failures rather than minor segmentation errors. Such failures are clinically unacceptable because they may adversely impact clinical decision-making if not detected during clinical review. Compared with the previously reported results on the TrueBeam datasets, the reduced geometric accuracy of PACS on the Ethos dataset, although not statistically significant, suggests potential limited generalizability across institutions and imaging platforms. These findings indicate that while the pre-trained model retained reasonable segmentation capability on independent datasets, differences in image characteristics between training and deployment environments can substantially affect the model performance. Adaptation of the model using Ethos-based CBCT data may therefore be necessary to improve robustness and generalizability before clinical deployment on such systems.

Dosimetric evaluation was also performed to assess the dosimetric impact of using contours generated by the PACS model. Although PACS demonstrated closer dose agreement with the reference contour compared with Rigid-Reg, significant reductions in GTV Dmean and D98% were still observed, suggesting that the segmentation errors were primarily associated with contour displacement rather than simple under-segmentation. Such positional shifts are clinically concerning because they may misrepresent the true target location and potentially lead to inappropriate treatment decisions. These dosimetric findings further reinforce the need for careful validation and adaptation of AI models to account for domain shifts before clinical implementation.

Regarding the esophagus segmentation, PACS did not demonstrate a significant improvement compared with simple rigid propagation, and no consistent pattern was observed in the performance differences between the two methods (table 2 and supplementary figure S2), highlighting the challenges of segmenting low-contrast structures and the limited benefit of PACS on this evaluation dataset. Compared with originally reported results on TrueBeam datasets, significantly degraded DSC and HD95 were observed, indicating degraded model performance when directly applying the model to a completely different clinical setting. However, the dosimetric parameters for esophagus, including Dmean and V32Gy, did not show significant deviation from the reference when using different contours. This may be because the esophagus is typically located far from the tumor and outside regions of steep dose gradients, such that contour mismatches do not result in substantial changes in dosimetric parameters.

As an external evaluation, several limitations regarding the dataset and evaluation strategy should be acknowledged. First, the clinical reference contours were initially generated using an automated ‘warm start’ before being modified by physicians, which may introduce an inherent automation bias. However, these contours reflect the actual clinical decisions made during routine adaptive treatment and therefore provide a clinically relevant reference for evaluating model performance. Second, focusing exclusively on the intra-ring region limits our ability to evaluate the model’s global segmentation accuracy. However, this localized approach was intentionally selected to prioritize clinical relevance and evaluation quality. Incorporating non-clinically approved anatomical regions as reference would compromise dataset integrity and undermine our primary goal of validating clinical acceptability. In addition, from a clinical dosimetry perspective, the segmentation accuracy of OARs far away from the target volume becomes less critical due to these structures residing in low-dose regions, further justifying our localized evaluation approach. Third, this study focused on geometric and dosimetric metrics to assess model generalizability. Future studies should incorporate additional clinical workflow assessments, including physician editing time, contour acceptability, and user experience, to further evaluate the practical benefits of AI-assisted segmentation in online ART. The impact of AI-generated contours on treatment plan re-optimization is also worth investigating, particularly for models capable of segmenting both target volumes and multiple OARs. Such evaluations would help determine whether automated segmentation translates into meaningful benefits in the downstream adaptive planning process. Another limitation for this study is the relatively small number of patients included. Although each patient contributed five CBCT scans acquired on different treatment days with expected anatomical changes, scans from the same patient remain partially dependent and do not represent fully independent samples. Furthermore, due to the use of contour ring, only 26 scans from 6 patients were available for the esophagus evaluation. Therefore, the current evaluation is limited in terms of inter-patient anatomical diversity. The mixed-effects model has been used to account for intra-patient correlation. Inclusion of a larger patient cohort would improve the generalizability of the findings, enable more comprehensive evaluation, and facilitate the identification of patterns associated with segmentation failures.

In conclusion, the performance of the PACS model trained on TrueBeam CBCTs was limited when evaluated on Ethos data, particularly for the esophagus that exhibits lower soft tissue image contrast. This performance gap likely reflects the domain shift between patient populations, clinical workflows, and imaging systems, which underscores the importance of incorporating multi-domain training data or domain adaptation strategies (Guan and Liu 2021) to improve the robustness of auto-segmentation models across different institutions. Furthermore, to facilitate the clinical implementation of DL-based auto-segmentation, developing systems to monitor input images and predict segmentation performance could be valuable.

## Data Availability

The data cannot be made publicly available upon publication because they contain sensitive personal information. The data that support the findings of this study are available upon reasonable request from the authors. Supplementary data 1 available at https://doi.org/10.1088/2057-1976/aeaf5c/data1.
